# Marine-derived protein kinase inhibitors for neuroinflammatory diseases

**DOI:** 10.1186/s12938-018-0477-5

**Published:** 2018-04-24

**Authors:** Chong Ning, Hui-Min David Wang, Rong Gao, Yu-Chia Chang, Fengqing Hu, Xianjun Meng, Shi-Ying Huang

**Affiliations:** 1 College of Light Industry, Liaoning University, Shenyang, 110036 China; 2 Graduate Institute of Biomedical Engineering, National Chung Hsing University, Taichung, 40227 Taiwan; 3 College of Oceanology and Food Science, Quanzhou Normal University, Quanzhou, 362000 China; 4 Yangtze Delta Region Institute of Tsinghua University, Zhejiang, 314006 China; 5 Jiaxing Deqin Biotechnology Co., Ltd, Zhejiang, 314006 China; 6 Greenhouse Systems Technology Center, Central Region Campus, Industrial Technology Research Institute, Nantou, 540 Taiwan; 7 College of Food Science, Shenyang Agricultural University, Shenyang, 110866 China; 8 Fujian Province Key Laboratory for the Development of Bioactive Material from Marine Algae, Quanzhou, 362000 China; 9 Key Laboratory of Inshore Resources Biotechnology (Quanzhou Normal University) Fujian Province University, Quanzhou, 362000 China

**Keywords:** Neuroinflammation, Protein kinase inhibitors, Marine, Glia, Immune cells, In vivo

## Abstract

Neuroinflammation is primarily characterized by overexpression of proinflammatory mediators produced by glial activation or immune cell infiltration. Several kinases have been shown to be critical mediators in neuroinflammation. One of the largest groups of kinases is protein kinases, which have been the second most studied group of drug targets after G-protein-coupled receptors. Thus far, most of the approved kinase inhibitor drugs are adenosine triphosphate-competitive inhibitors with various off-target liabilities because of cross-reactivities; however, marine-derived compounds provide opportunities for discovering allosteric kinase inhibitors. This review summarizes the potential of marine-derived protein kinase inhibitors in the field of neuroinflammatory diseases, such as Parkinson disease, Alzheimer disease, multiple sclerosis, and pain. The previous studies from 1990 to 2017 in this review have shown that marine-derived protein kinase inhibitors have great potential to elicit anti-neuroinflammatory or neuroprotective responses in in vitro and in vivo models of neuroinflammatory diseases. This suggests that further exploration and investigation of these marine-derived protein kinase inhibitors on neuroinflammatory diseases are warranted. Therefore, this review may inspire further discovery of new protein kinase inhibitors from a marine origin and additional neuroscience studies focusing on these valuable marine-derived protein kinase inhibitors.

## Neuroinflammatory diseases

Basically, inflammation could remove detrimental stimuli or initiate tissue healing, and therefore inflammation is a necessary and protective physiologic response to injury or infection. Similarly to inflammation, a process in response to nervous system injury is termed neuroinflammation. However, the prolonged neuroinflammation exceeds the bounds of physiological control and generates deleterious effects, including proinflammatory signaling pathways, oxidative stress, and even neuron death [1, 2]. The types of activated cells that could cause neuroinflammation comprise the glial cells [including Schwann cells and satellite glial cells in the peripheral nervous system (PNS), and microglia, astrocytes, and oligodendrocytes in the central nervous system (CNS)], and the immune cells (including resident mast cells and macrophages as well as infiltrating neutrophils and T cells) [3]. In other words, neuroinflammation is characterized by immune cell infiltration or glial cell activation, with inflammatory mediator production, in the PNS and CNS [4]. There are several major neuroinflammatory diseases, such as Parkinson disease (PD) [5], Alzheimer disease (AD) [6], multiple sclerosis (MS) [7], pain [4, 8], epilepsy [9], HIV-1-associated neurocognitive disorders (HANDs) [10], Huntington disease (HD) [11], and brain ischemia [12]. The human genome has approximately 519 kinases [13]. One of the largest groups of kinases is protein kinases, which catalyze key phosphorylation pathways that regulate most aspects of cell life and have become the second most studied group of drug targets after G-protein-coupled receptors [13]. Inhibition of protein kinases are anticipated to be a source of potential therapeutic targets for human neuroinflammatory diseases, such as glycogen synthase kinase (GSK)-3β and cyclin dependent kinase (CDK) in PD [14] and AD [15]; c-Jun N-terminal kinase (JNK), extracellular signal-regulated kinase (ERK), and p38, subgroups of mitogen activated protein kinases (MAPKs), in PD [14], AD [15], and pain [16, 17]; FMS-like tyrosine-3 (FLT-3) and Janus kinase (JAK) in MS [18, 19]; protein kinase C (PKC) in pain [20]; tropomyosin-related kinase (Trk) in epilepsy [21]; Ca^2+^/calmodulin (CaM)-dependent protein kinase (CaMK) in ischemia [22]. Most kinase-targeted drugs and related studies about potential kinase targets focus on non-CNS disorders and neurological tumors, although there are two kinase inhibitors for neuroinflammatory diseases: lithium for AD by targeting GSK-3 (clinical phase II); dilmapimod (SB-681323) for neuropathic pain by targeting p38α (clinical phase II) [23].

## Protein kinase inhibitors from a marine origin for neuroinflammatory diseases

Almost all of the current therapeutic indications of protein kinase inhibitors are for neoplastic diseases. The treatment approval of tofacitinib for rheumatoid arthritis (in 2012) and nintedanib for idiopathic pulmonary fibrosis (in 2014), which implies that an expanded therapeutic repertoire of protein kinase inhibitors will grow in the future [24, 25]. About one-quarter of the druggable genome are kinases [26]. These findings pave a path forward for repurposing existing clinical and preclinical protein kinase inhibitors safely and efficiently as potential treatment for non-cancer diseases. On the other hand, the number of small molecule protein kinase inhibitors approved by the Food and Drug Administration (FDA) has increased at a rate of 2–4 per year, and no sign shows that this growth trend will slow down [24, 25]. Thus far, most of the approved kinase inhibitor drugs are adenosine triphosphate-competitive inhibitors with various off-target liabilities because of cross-reactivities. However, marine-derived compounds could provide additional opportunities for discovering allosteric kinase inhibitors [13]. Twelve protein kinase inhibitors from a marine origin have entered preclinical and clinical trials: four kinase inhibitors (i.e., midostaurin, meisoindigo, lestaurtinib, enzastaurin) have entered phase III clinical studies, four kinase inhibitors (i.e., UCN-01, kahalalide F, CEP-2563, CEP-1347) have entered phase II clinical studies, one kinase inhibitor (i.e., isokahalalide F) has entered phase I clinical studies, and three kinase inhibitors (i.e., staurosporine, variolin B, fascaplysin) have entered preclinical investigations [13]. Whereas the therapeutic area of CEP-1347 in clinical studies was PD, the 12 other protein kinase inhibitors from a marine origin in clinical and preclinical studies were cancer [13]. Although neuroinflammatory diseases and cancer differ in several respects, they share basic mechanisms such as inflammatory processes; thus, protein kinase inhibitors may play a crucial role in drug development in both neuroinflammatory diseases and cancer. Chronic inflammatory condition increases the risk of cancers, and potent epidemiological evidence shows that non-steroidal antiinflammatory drugs (NSAIDs), especially aspirin, are chemopreventive agents [27]. Moreover, many anti-cancer agents have been also used for treating inflammatory diseases (such as rheumatoid arthritis) [28]. Several of the aforementioned marine-derived protein kinase inhibitors have been investigated for neuroinflammatory diseases by using in vitro and in vivo systems (Table 1).

**Table 1:** Clinical and preclinical studies on marine-derived protein kinase inhibitors for neuroinflammatory diseases

Status for the original application	Compound	Source	Target	Models of neuroinflammatory diseases
Phase III for AML	Lestaurtinib (CEP-701)	Derived from K-252a from marine actinomycete	FLT-3, JAK-2, Trk-A, Trk-B, Trk-C	MS: in vivo [29, 30] Epilepsy: in vivo [31]
Phase III for glioblastoma and diffuse large B-cell lymphoma	Enzastaurin (LY317615)	Derived from staurosporine	PKCβ, GSK-3β	MS: in vivo [32] Pain: in vivo [33]
Phase II for PD	CEP-1347 (KT7515)	Derived from K-252a	JNKs	PD: patients [34]; in vivo [35] AD: in vitro [36, 37] HANDs: in vivo [10] HD: in vivo [38, 39] Ischemia: in vivo [40]
Preclinical for cancer	Staurosporine (AM-2282)	From marine organisms such as prosobranch mollusk, flatworm, and ascidians	PKC, JAK-2, CaMKIII	PD: in vitro [41] AD: in vitro [42] Pain: in vivo [43] Epilepsy: in vivo [44] Ischemia: in vivo [45, 46]
Preclinical for cancer	Fascaplysin	From marine sponge	CDK-4	AD: in vitro [47]

*AD* Alzheimer disease, *AML* acute myelogenous leukemia, *CaMK* Ca^2+^/calmodulin (CaM)-dependent protein kinase, *CDK* cyclin dependent kinase, *FLT-3* FMS-like tyrosine-3, *HANDs* HIV-1-associated neurocognitive disorders, *HD* Huntington disease, *GSK* glycogen synthase kinase, *JAK* Janus kinase, *JNK* c-Jun N-terminal kinase, *MS* multiple sclerosis, *PD* Parkinson disease, *PKC* protein kinase C, *Trk* tropomyosin-related kinase

## Lestaurtinib

**Fig. 1:**
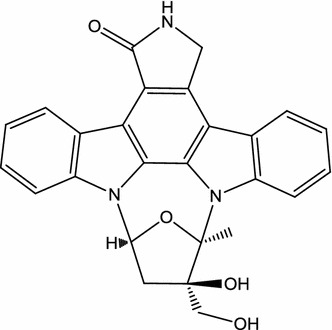
Chemical structure of lestaurtinib

Lestaurtinib (CEP-701; Fig. 1), a polyaromatic indolocarbazole alkaloid derived from K-252a, is orally bioavailable in phase III clinical studies on acute myelogenous leukemia (AML) (Cephalon, Frazer, PA, USA) [48]. In 1985, Kase et al. isolated K-252a (previously named K-252; an indolocarbazole) from a culture broth of a marine actinomycete *Nocardiopsis* sp., *Nonomuraea longicatena*. Subsequently, in 1986, Kase et al. reported that K-252a is a potent inhibitor of protein kinase C and inhibits calmodulin [49]. In 1997, Kaneko et al. also reported that K-252a have broad serine/threonine and tyrosine kinase inhibitory activity, such as trk A, protein kinase C1, protein kinase A, and myosin light chain kinase [50]. Although, in relapsed/refractory (R/R) *FLT3*-internal tandem duplication (ITD) AML patients in 2011 [51] and during front-line consolidation in the MRC AML15 trial in 2014 [52], lestaurtinib associated with chemotherapy did not improve outcomes. In 2017, Knapper et al. reported that the addition of lestaurtinib to standard chemotherapy for newly diagnosed *FLT3*-mutated AML yielded no overall clinical benefit [53]. Peripheral treatment of mice with lestaurtinib (20 mg/kg, twice daily) led to a substantial improvement in locomotor function and myelin preservation in the course of established experimental autoimmune encephalomyelitis (EAE) induced by myelin oligoglycoprotein (MOG_35–55_), an in vivo model for MS [29]. Moreover, in EAE mice, a decrease in the number and activation states of both peripheral dendritic cells and microglia in the CNS participated in the therapeutic effects of lestaurtinib [30]. Recently, intraperitoneal (i.p.) lestaurtinib (3 mg/kg, twice daily) has been reported to attenuate hypoxic seizure (HS)-induced seizure susceptibility in rat pups, likely through its inhibition of tropomyosin receptor kinase B [31].

## Enzastaurin

**Fig. 2:**
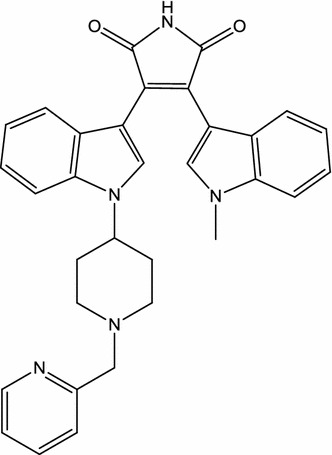
Chemical structure of enzastaurin

Enzastaurin (LY317615; Fig. 2), an oral sugar ring modified analogue of staurosporine [13], has been examined in phase III trials for glioblastoma and diffuse large B cell lymphoma [54]. The combination of enzastaurin (twice daily oral administration at 75 mg/kg) with local irradiation attenuates osteolytic lesions and both spontaneous and movement-evoked pain behaviors in mice caused by metastatic breast cancer in bone, an in vivo model for bone cancer pain [33]. Oral treatment of mice with ongoing EAE with enzastaurin (50 mg/kg, once daily) ameliorates neuroinflammation (CNS infiltration of myelin-specific T cells), demyelination, axonal damage, and clinical symptoms (limb weakness or paralysis) [32].

## CEP-1347

**Fig. 3:**
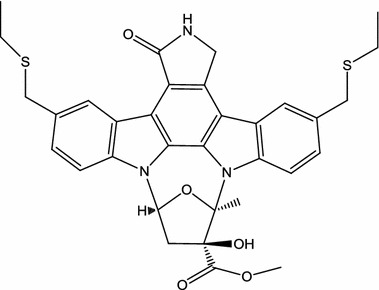
Chemical structure of CEP-1347

A compound CEP-1347 (KT7515; Fig. 3), originally discovered through a program researching the neurotrophic properties of semisynthetic derivatives of K-252a [55], and CEP-1347 is inhibitor of the JNK signal-transduction pathway upstream by targeting the level of the mixed lineage kinases (MLKs, including MLK1, MLK2,and MLK3) [56]. In 1999, Saporito et al. reported that subcutaneous (s.c.) administration of CEP-1347 (0.3 mg/kg/d) attenuated 1-methyl-4-phenyl tetrahydropyridine-mediated nigrostriatal dopaminergic degeneration in a mouse model of PD [35]. In early PD patients, CEP-1347 [in dosages of 10 mg twice daily (*n* = 205), 25 mg twice daily (*n* = 212), or 50 mg twice daily (*n* = 198)] fails to delay disability [34]. The following studies on CEP-1347 for other neuroinflammatory diseases are described as follows: In an in vitro model of AD, CEP-1347 (300 nM) promotes survival and blocks activation of a Jun-N terminal kinase pathway associated with β-amyloid (Aβ)-induced cortical neuron apoptosis [36]. In the in vitro models of AD in another previous study, CEP-1347 (100–300 nM) also effectively protected Aβ-evoked-death in both PC12 cells and sympathetic neurons [37]. CEP-1347 (500 nM) inhibited mutant huntingtin-associated neurotoxicity in an in vitro HD model, and CEP-1347 (s.c.; 0.5 mg/kg/day) reduced the decline in motor performance and restored cortical brain-derived neurotrophic factor BDNF levels in R6/2 transgenic mice of the HD model [38]. Four hours after a single s.c. injection, CEP-1347 (1 mg/kg) was reported to increase brain-derived neurotrophic factor (BDNF) levels in blood in R6/2 mice model of HD through an increased transcription from BDNF promoter III [39]. Using in vitro models of HANDs, Eggert et al. demonstrated that CEP-1347 (220 nM) elicits an antiinflammatory phenotype in HIV-1-infected primary human monocyte-derived macrophages (MDM), thereby reducing the neurotoxicity mediated by HIV-1-infected MDM for primary murine cortical neurons [10]. In addition, the in vivo data from a mouse model for HIV-1 encephalitis showed that CEP-1347 (i.p.; 1.5 mg/kg/day) expedites neuronal survival and reduces microglial activation and dendritic damage in the brain [10]. In addition to the effects of CEP-1347 on the neurons and microglia, there is also a report about the effects of CEP-1347 on astrocytes. In the primary murine cortical astrocyte inflammation of an in vitro model, CEP-1347 is an inhibitor of astrocyte nitric oxide (NO) release (the IC50 values of CEP-1347 were 90 ± 10 nM), and CEP-1347 blocks the expression of inducible NO synthase (iNOS) and cyclooxygenase-2 (COX-2) at the transcriptional level [57]. In a neonatal rat model of hypoxia–ischemia, s.c. CEP-1347 (1 mg/kg, once daily) has protective effects, which is related to reduced apoptosis [40].

## Staurosporine

**Fig. 4:**
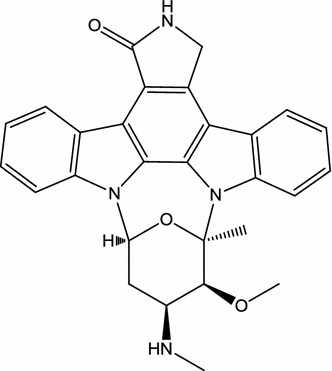
Chemical structure of staurosporine

In 1977, during a search for new alkaloids present in *actinomycetes*, Omura et al. isolated AM-2282 (renamed staurosporine; Fig. 4) from *Streptomyces staurosporeus* [58]. Staurosporine has been isolated from some marine organisms such as the prosobranch mollusk, flatworm, and ascidians [59]. In 1990, Hara et al. showed that topical injection into the CA_l_ subfield of the hippocampus of staurosporine (10 ng) administered 30 min before ischemia prevented neuronal death in gerbil and rat ischemia models [45]. Moreover, Ohno et al. indicated that staurosporine administered (i.p.; 0.03 mg/kg) immediately after blood flow reperfusion significantly reduced the impairment of working memory in rats following transient forebrain ischemia [46]. Both seizure-induced damage to hippocampal neurons and associated visuospatial memory deficits were significantly reduced in rats administered staurosporine (s.c. injections of 4 μg/kg/day) prior to kainic acid administration [44]. In addition to these in vivo studies, in in vitro models of AD, staurosporine (100 pM) can protect cultured rat hippocampal neurons against Aβ toxicity or iron-induced injury [42]. Moreover, intradermal injection of staurosporine (500 ng) reduced the mechanical hyperalgesia in streptozotocin-induced diabetic rats but did not alter thresholds in normal rats [43]. On the other hand, one of the pathological characteristics in the early stages of PD is axonal degeneration of dopaminergic neurons, and therefore promotion of axonal outgrowth of the remaining dopaminergic neurons results in the recovery of the nigrostriatal pathway [41]. Through AMP-activated protein kinase (AMPK)/mammalian target of rapamycin (mTOR) signaling pathway, staurosporine (10 nM) induces dopaminergic neurite outgrowth in mesencephalic primary cultures [41]. However, staurosporine is excessively toxic for clinical development [13]. Through improved toxicity profiles, several staurosporine analogues have advanced to various stages of clinical development, such as midostaurin (clinical phase III), lestaurtinib (clinical phase III), enzastaurin (clinical phase III), edotecarin (not an inhibitor of protein kinases; clinical phase III), becatecarin (not inhibitor of protein kinases; clinical phase III), UCN-01 (clinical phase II), CEP-2563 (clinical phase II), and CEP-1347 (clinical phase II) [13].

## Fascaplysin

**Fig. 5:**
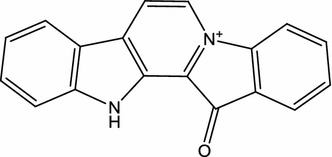
Chemical structure of fascaplysin

Fascaplysin (Fig. 5), a bis-indole alkaloid, was isolated from a marine sponge *Fascaplysinopsis* Bergquist sp. [60]. Fascaplysin is a specific kinase inhibitor for CDK 4 [61]. In 2013, Sanphui et al. demonstrated that fascaplysin chloride (0.4 μM) protected rat neuronal pheochromocytoma 12 cells from death induced by nerve growth factor deprivation, an in vitro model of AD [47].

## Future advantages and challenges for marine-derived protein kinase inhibitors

**Fig. 6:**
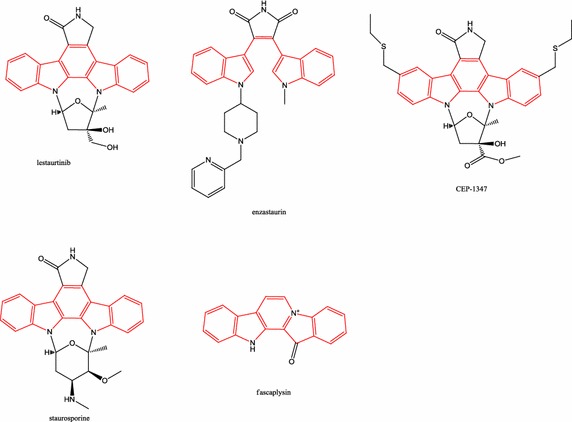
The core skeleton of five marine-derived protein kinase inhibitors for neuroinflammatory diseases. The color of the core skeleton is red

Figure 6 shows the core skeleton of five marine-derived protein kinase inhibitors for neuroinflammatory diseases. It would further be expected that the analogues of 11,12-dihydroindolo[2,3-a]carbazole and *N*-substituted β-carboline class of fascaplysin-inspired compounds might have great potential to elicit antineuroinflammatory or neuroprotective responses in models of neuroinflammatory diseases. The CDK inhibitor (including fascaplysin) could be potential antiinflammatory and pro-resolution agents, such as R-roscovitine have shown potential antiinflammatory effects that could influence the resolution of inflammation [62]. In 1996, Smith-Swintosky et al. considered that low-molecular-weight lipophilic alkaloids (K252 compounds and staurosporine) can pass the blood–brain barrier (BBB) [44], and this hypothesis is supported by the aforementioned studies conducted in vivo. The ability of these marine-derived protein kinase inhibitors to pass the BBB is an advantage for future clinical uses for both neuroinflammatory diseases and tumors in the CNS. Specifically, at present, the therapeutic area of these protein kinase inhibitors from a marine origin in clinical and preclinical studies is peripheral cancer. The topic of these marine-derived protein kinase inhibitors on CNS tumors might be another worthwhile direction for further research. In addition, there were several patents about application of these marine-derived protein kinase inhibitors or their derivatives, including lestaurtinib for the treatment of neurodegenerative diseases [63] and the treatment or prevention of pain disorders [64], enzastaurin for the treatment of neurological diseases [65], and bis-N-substituted derivatives of staurosporine for the treatment of neurological disorders [66, 67]. Recent projects have begun to identify non-kinase targets for kinase inhibitors [68], which could be the third possible future research directions of these marine-derived protein kinase inhibitors. On the other hand, because the problem of a continuous supply of natural marine products is a major challenge in conducting preclinical and clinical trials on marine drugs [69, 70], few marine-derived protein kinase inhibitors for neuroinflammatory diseases have been approved by the FDA and few studies have been conducted in vivo [71]. To overcome the supply problem of natural marine products, several strategies, such as (1) total chemical synthesis (2) semisynthetic production, (3) fermentation, (4) sampling strategies, (5) nanoscale NMR for structure elucidation, and (6) biotechnology [69, 70, 72], are being developed. Another major challenge for natural marine products is target identification [70]. Although the molecular target-based approach (i.e., reverse pharmacology) for drug discovery has been widely adopted in the past 25 years, phenotypic-based screening strategies (i.e., forward pharmacology) have become the foundation of pharmaceutical drug discovery [73]. Based on phenotypic-based screening strategies, several marine-derived compounds might reduce in vitro (Table 2) or in vivo (Table 3) neuroinflammatory processes. Grosso et al. have outlined bioactive marine compounds with anti-neuroinflammatory activity [71]. Barbosa et al. also have summarized bioactive compounds from macroalgae for several neuroinflammatory diseases [74]. Although target identification for phenotypic-based screening strategies is complex and time consuming [73], further target identification of these marine-derived compounds is warranted. The third major challenge for natural marine products, particularly for marine-derived protein kinase inhibitors, is identifying new drug targets from biomedical research [73]. After reviewing 989 FDA-approved drugs with human targets from the DrugBank database, Rask-Andersen et al. indicated only 435 effective drug targets [75], although 30,000 human genes create approximately 90,000 proteins [76]. Zheng et al. asserted that failures in discovery and validation of new targets have partially contributed to the substantial decrease in the quantity of newly approved drugs in the past decade [73].

**Table 2:** The marine-derived extracts or compounds with in vitro anti-neuroinflammatory activity

Extracts or compounds	Marine source	Therapeutic area	Possible molecular pathway
Methanol extracts	Marine green algae *Ulva conglobata*	Neuroinflammation	Inhibition of iNOS and COX-2 [77]
Alginate-derived oligosaccharide	Various brown algae	AD	Inactivation the TLR4-NF-κB signaling pathway [78]
Dieckol	Brown algae *Ecklonia cava*	Neuroinflammation	Downregulation of ERK, Akt and NADPH oxidase-mediated pathways [79]
Floridoside	Red algae *Laurencia undulata*	Neuroinflammation	Inhibition of p38 and ERK [80]
Phlorofucofuroeckol B	Brown algae *Ecklonia stolonifera*	Neuroinflammation	Inhibition of IκB-alpha/NF-κB and Akt/ERK/JNK pathways [81]
Aurantiamide acetate	Marine fungus *Aspergillus* sp. SF-5921	Neuroinflammation	Inhibition of NF-κB, JNK, and p38 [82]
Citreohybridonol	Marine fungus *Toxicocladosporium* sp. SF-5699	Neuroinflammation	Inhibitory effect on the NF-κB and p38 pathways [83]
Sinuleptolide	Soft coral *Sinularia kavarattiensis*	Neuroinflammation	Inhibition of IL-1β, IL-6, IL-8, IL-18, and TNF-α [84]

*AD* Alzheimer disease, *COX-2* cyclooxygenase-2, *ERK* extracellular signal-regulated kinase, *IκB* inhibitor of NF-κB, *IL* interleukin, *iNOS* inducible nitric oxide synthase, *JNK* c-Jun N-terminal kinase, *NADPH* nicotinamide adenine dinuclelotide phosphate, *NF-κB* nuclear factor κB, *TLR4* toll-like receptor 4, *TNF-α* tumor necrosis factor-α

**Table 3:** The marine-derived extracts or compounds with in vivo anti-neuroinflammatory activity

Extracts or compounds	Marine source	Therapeutic area	Possible molecular pathway
Ethanol extract	Marine microalgae *Nannochloropsis oceanica*	AD	Down-regulation of APP and BACE1 expression [85]
MS14	MS14 is a natural herbal-marine drug	MS	Inhibition of spinal LCN2 [86, 87] (MS14 consists of 90% *Penaeus latisculatus* (king prawn), 5% *Apium graveolens* (celery), and 5% *Hypericum perforatum* L. (St John’s wort).)
11-Dehydrosinulariolide	Soft coral *Sinularia flexibilis*	PD	In vitro: inhibition of NF-κB [88] In vivo: increase of DJ-1 expression [89]
Dihydroaustrasulfone alcohol	The synthetic precursor of austrasulfone from the Soft coral *Cladiella australis*	MS and pain	Inhibition of iNOS and COX-2 based on in vitro data [90]
Capnellene	Soft coral *Capnella imbricate*	Pain	Inhibition of spinal COX-2 [91]
Lemnalol	Soft coral *Lemnalia cervicorni*	Pain	Inhibition of spinal TNF-α [92]
Flexibilide	Soft coral *Sinularia flexibilis*	Pain	Inhibition of spinal iNOS [93]
DHA	Fish	Pain	Inhibition of spinal p38 [94]
Xyloketal B	Marine fungus *Xylaria* sp. (strain no. 2508)	Ischemia	Inhibition of brain caspase-3 and Bax [95]
Polyphenols	Brown algae *Ecklonia cava*	Ischemia	Inhibition of cytosolic calcium based on in vitro data [96]

*AD* Alzheimer disease, *BACE1* beta-secretase 1, *COX-2* cyclooxygenase-2, *DHA* docosahexaenoic acid, *iNOS* inducible nitric oxide synthase, *LCN2* Lipocaline2, *MS* multiple sclerosis, *NF-κB* nuclear factor κB, *PD* Parkinson disease, *TNF-α* tumor necrosis factor-α

## Summary

The studies from 1990 to 2014 in this review have demonstrated that marine-derived protein kinase inhibitors (i.e., lestaurtinib, enzastaurin, CEP-1347, staurosporine, and fascaplysin) have great potential to elicit anti-neuroinflammatory or neuroprotective responses in in vitro and in vivo models of human neuroinflammatory diseases. This suggests that further exploration and investigation of these marine-derived protein kinase inhibitors on neuroinflammatory diseases are warranted. Therefore, the present review may inspire further discovery of new protein kinase inhibitors from a marine origin and neuroscience researchers to perform additional studies focusing on these valuable marine-derived protein kinase inhibitors.
